# Can current molecular tests help in the diagnosis of indeterminate thyroid nodule FNAB?

**DOI:** 10.20945/2359-3997000000081

**Published:** 2018-10-01

**Authors:** Carolina Ferraz

**Affiliations:** 1 Irmandade da Santa Casa de Misericórdia de São Paulo Irmandade da Santa Casa de Misericórdia de São Paulo Departamento de Medicina Divisão de Endocrinologia São Paulo SP Brasil Unidade de Doenças da Tireoide, Divisão de Endocrinologia, Departamento de Medicina, Irmandade da Santa Casa de Misericórdia de São Paulo, São Paulo, SP, Brasil

**Keywords:** Thyroid, nodules, molecular test, indeterminate samples

## Abstract

Approximately 15–30% of all thyroid nodules evaluated with fine-needle aspiration biopsy (FNAB) are classified as cytologically indeterminate. The stepwise unraveling of the molecular etiology of thyroid nodules has provided the basis for a better understanding of indeterminate samples and an opportunity to decrease diagnostic surgery in this group of patients. Over the last 15 years, several studies have tested different methodologies to detect somatic mutations (by polymerase chain reaction and next-generation sequencing, for example), and to identify differentially expressed genes or microRNA, aiming at developing molecular tests to improve the presurgical diagnosis of cytologically indeterminate nodules. In this review, we will provide an overview of the currently available molecular tests and the impact of mutation testing on the diagnosis of thyroid cancer. We will also review current published data and future perspectives in molecular testing of thyroid nodule FNAB and describe the current Brazilian experience with this diagnostic approach. Based on currently available data, especially for countries outside the US-Europe axis, a rational use of these tests must be made to avoid errors with regard to test indication and interpretation of test outcomes. In addition to clinical, radiological, and cytological features, we still need to determine local malignancy rates and conduct more independent validation and comparative performance studies of these tests before including them into our routine approach to indeterminate FNAB.

## INTRODUCTION

Even though the introduction of fine-needle aspiration biopsy (FNAB) has improved the selection of suspicious nodules for surgery (1), approximately 15% to 30% of all thyroid nodules undergoing FNAB are classified as cytologically indeterminate, which includes lesions of undetermined significance/follicular lesion of undetermined significance (AUS/FLUS; Bethesda III) or follicular neoplasm/suspicious for follicular neoplasm (FN/SFN; Bethesda IV) (2,3). The stepwise unraveling of the molecular etiology of thyroid nodules has provided the basis for a better understanding of cytologically indeterminate nodules and a chance to reduce diagnostic surgery in this scenario. Because immunocytological markers have failed to show enough specificity and sensitivity, improved molecular testing for common somatic mutations (*i.e., BRAF* and *RAS* point mutations, or *RET/PTC* and *PAX8/PPAR*γ rearrangements) and identification of gene and microRNA (miRs) expression classifiers have emerged as the most promising approaches.

During the last 15 years, several studies have tested different diagnostic methodologies to characterize thyroid cancer. The latest studies in this field have sought specific molecular markers to differentiate benign and malignant neoplasms and discriminate with increased sensitivity and specificity the different histotypes of thyroid cancer.

In this review, we will provide a critical overview of the current impact of mutation testing on the diagnosis of thyroid cancer, discussing current possibilities and future perspectives in molecular testing of thyroid nodule FNABs. Additionally, we will describe the current Brazilian experience with molecular testing of thyroid nodules deemed indeterminate on FNABs.

### Molecular testing from the beginning until now

After serum-based biomarkers like calcitonin and thyroglobulin were first described, they provided the impetus for future research in the discovery of biomarkers for the diagnosis of thyroid cancer (4). With advances in genomic and proteomic technologies, new biomarkers for thyroid cancer have emerged.

Studies have investigated the role of immunocytological markers like galectin-3 (5-7), HBME-1 (6-8), fibronectin-1, CITED-1, and cytokeratin-19 (6,7) in improving the differential diagnosis between benign and malignant thyroid nodules. However, these markers have been barely incorporated in daily routine diagnostics, mainly due to their different methodologies and considerable overlapping identification of follicular adenomas (FA) and differentiated thyroid carcinomas (DTC) (9,10). A further and important step has been achieved with the discovery of somatic mutations in about two-thirds of the papillary thyroid carcinomas (PTCs) and follicular thyroid carcinomas (FTCs), offering new perspectives for the classification and diagnosis of thyroid tumors (11).

**Figure 1:**
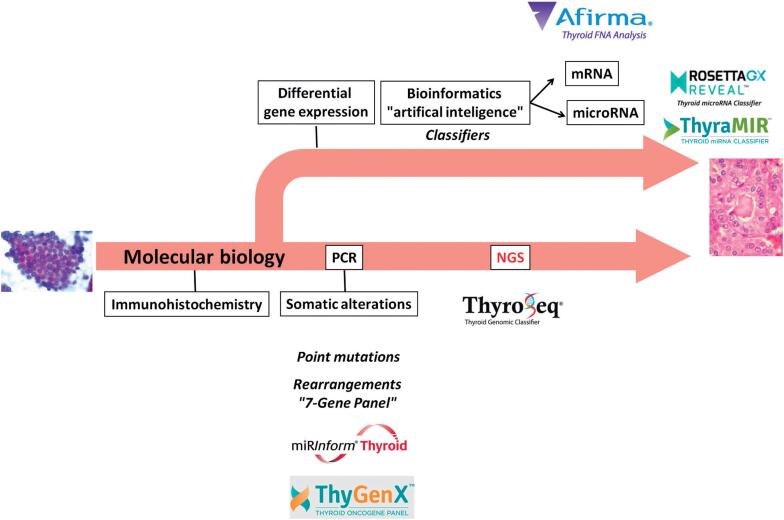
Commercially available tests and their technology over time.

Molecular testing for somatic mutations has become a promising approach and is currently the most studied molecular diagnostic method in FNAB (12,13). In most thyroid cancers, these mutations are mutually exclusive events, meaning, only one of these mutations is found in any particular cancer (14). When these mutations are used as independent biomarkers, their sensitivity and specificity are too low to be clinically relevant. However, a panel of mutations has been shown to improve both sensitivity and specificity rates (15). Nikiforov and cols. were the first group to report a gain in sensitivity (from 44% to 80%) and accuracy (from 93.3% to 97.4%) by analyzing a panel of *BRAF, RAS*, *RET/PTC*, and *PAX8/PPAR*γ mutations (15,16). Based on this evidence, the first commercially available test named “miRInform” (Asuragen, Inc., Austin, TX, USA) (Figure 1) was created in 2009. *BRAF, KRAS*, *HRAS, NRAS*, and chromosomal translocations resulting in *RET/PTC1, RET/PTC3*, and *PAX8*/*PPAR*γ fusions resulted in the development of a “7-Gene Panel.” This test was later replaced and is currently offered by Interpace Diagnostics (Parsippany, NJ, USA) as the ThyGenX test (Figure 1), with *PIK3CA* added to the panel.

Next-generation sequencing (NGS) technologies or massively parallel sequencing are related terms that describe a DNA sequencing technology that has revolutionized genomic research and emerged as a step up from the Sanger sequencing method. Briefly, NGS can be used to sequence entire genomes or be constrained to specific areas of interest, including all 22,000 coding genes (the entire exome) or small numbers of individual genes (17). The publication of the integrated genomic characterization of PTC by The Cancer Genome Atlas (TCGA) has reduced the fraction of PTC cases with unknown oncogenic driver from 25% to 3.5% (18), offering a high potential for molecular diagnostics.

The first targeted NGS panel customized for thyroid cancer was the ThyroSeq, which is commercially offered by CBLPath (Rye Brook, NY, USA) (Figure 1). In addition to the mutations detected by the 7-Gene Panel, other newly identified driver mutations in *PIK3CA, TP53*, *TSHR, PTEN*, *RET, AKT1*, *CTNNB1*, and *TERT*, as well as gene fusions involving *BRAF, RET*, *NTRK1, NTRK3*, *AKT1, PPARG*, and *THADA* have been added to the ThyroSeq panel (19,20).

As an alternative to mutation testing, the analysis of differentially expressed genes has emerged. In this line of thought, the definition of gene expression patterns of different types of thyroid tumors has been shown to be a promising approach. The role of bioinformatics and the use of artificial models can create computer algorithms and, thus, molecular classifiers differentiating FA and FTC/follicular variant PTC to improve the differential diagnosis of cytologically indeterminate FNABs (21).

Array technology has emerged as a powerful tool to assess the expression of a large number of genes. The Afirma Gene Expression Classifier (GEC) from Veracyte, Inc. (South San Francisco, CA, USA) embraces this approach by using microarray technology (Figure 1). A 167-gene classifier was developed, for which high sensitivity and negative predictive values (NPVs) have been reported (22).

Using the same rationale as that of the GEC, after the first miR was discovered, miR analyses gained an important place within the study of molecular markers (23-28). The most described (at least in three studies) differentially expressed upregulated miRs in benign *versus* malignant nodules are miR-221, miR-222, miR-146b, miR-21, miR-187, miR-197, and miR-181a (for a review on this topic, please refer to reference 29). ThyraMIR and RosettaGX Reveal are two miR panels that have been added to the market (Figure 1). The first test is performed on fresh FNAB material, while the second is performed on slide material.

Five tests are currently commercially available for thyroid FNABs: the new Afirma Genomic Sequencing Classifier (GSC; Veracyte, Inc., South San Francisco, CA, USA), the new version of ThyroSeq v3 (CBLPath, Inc, Rye Brook, NY, and University of Pittsburgh Medical Center, Pittsburgh, PA, USA), ThyGenX/ThyraMIR (Interpace Diagnostics, LLC, Parsippany, NJ, USA), ThyroPrint (GeneproDX, Santiago, Chile) and Mir-THYpe (ONKOS Diagnósticos Moleculares LTDA, Ribeirão Preto, Brazil). The last two tests have not been certified by the College of American Pathologists yet, and the last is currently only available in Brazil. Recently, RosettaGX Reveal (Rosetta Genomics, Inc., Philadelphia, PA, USA) has been withdrawn from the market for undeclared reasons. However, the test became commercially available again during the period in which this review was written, and will then be described in the text.

### How to evaluate the quality of a molecular test

**Figure 2:**
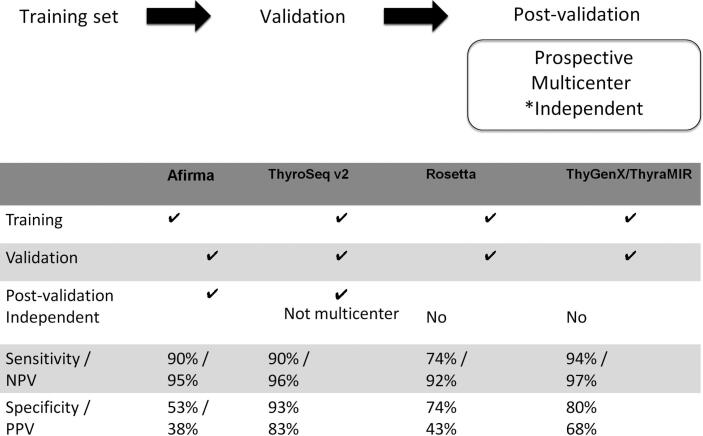
Steps for the commercialization of a molecular test.

Before a test becomes commercially available, three steps are usually followed (Figure 2): the first step is to identify and define the mutation panel or the classifier using a training set. The second step is to validate the panel/classifier on a validation set, and, finally, the third and most important step is the validation of the panel/classifier in a prospective, multicenter, and independent study.

To date, most commercially available tests have been evaluated following these steps. However, the RosettaGX Reveal and the ThyGenX/ThyraMIR have not been evaluated in independent studies. Moreover, since there are no independent publications for the new Afirma-GSC and ThyroSeq v3 until this review was written, we will discuss the performance of the latest version of each test and their validation studies (Figure 2).

The most comprehensively tested and evaluated commercial test is the 7-Gene Panel. After the first description of the *BRAF* mutation by Kimura and cols. in 2003 (14), with the recognition of the oncogenic role of the BRAF V600E mutation in approximately 58–69% of all PTCs, molecular testing for somatic mutations became an immediate approach and was the most promising molecular diagnostic tool in FNAB (15). However, genetic testing for BRAF V600E alone for the detection of PTCs is inadequate for clinical decision making, due to its low sensitivity (60%) for detecting PTCs (30). The highest sensitivity for the identification of thyroid cancers within the cytologically indeterminate category (63.7%) has been achieved by the analysis of a panel of mutations (15). The commercially available 7-Gene Panel miRInform was tested by Valderrabano and cols., who detected a mutation in 16% of 109 indeterminate nodules tested, all in Bethesda IV samples. Sensitivity and specificity in Bethesda IV specimens were 63% and 86% respectively, yielding an NPV and a positive predictive value (PPV) of 75% and 77%, respectively, and performing worse than it had done in the original study (31).

In a first independent evaluation, the Afirma-GEC (32) could only identify 27% of the benign nodules instead of the 53% described by Alexander and cols. (22), and only 17% of the suspicious thyroid nodules could be confirmed to be malignant instead of the reported 38%. Therefore, the clinical applicability of the classifier showed to be questionable after this first validation study, as the NPV of the Afirma-GEC was lower than that reported by Alexander and cols. Most of these differences can be explained by the fact that PPVs and NPVs vary according to the prevalence of thyroid carcinoma in the indeterminate FNAB categories analyzed. The higher the prevalence of thyroid cancer in an indeterminate category, the higher the PPVs; and the lower the prevalence of thyroid cancer in an indeterminate category, the higher the NPVs. Twenty additional validation studies have shown a wide variability in sensitivity (75% to 100%), specificity (5% to 53%), PPV (13% to 100%), and NPV (20% to 100%) (33). In addition to the prevalence of thyroid carcinoma in the analyzed indeterminate FNAB categories, the wide variation among the reported diagnostic values can also be explained by different defining characteristics of the study populations, such as the institutional prevalence of malignancy, sample size, Bethesda type and proportions of each Bethesda type included, the study definition of a “benign” tumor, and the predominance of Hürthle cell (HC) tumors (31).

The performance of the ThyroSeq v2 panel has been evaluated in eight single-institution studies (33). In the original publication, which first validated the ThyroSeq v2, the test demonstrated a sensitivity of 90%, specificity of 93%, NPV of 96%, and PPV of 83%. Additional validation of the panel in eight studies has shown a wide range of values, including sensitivity of 40–100%, specificity of 56–93%, PPV of 13–90%, and NPV of 48–97% (for details, please refer to reference 33); these values are also lower than those first reported by Nikiforov and cols. (19,20).

ThyGenX/ThyraMIR, RosettaGX Reveal, ThyroPrint, and Mir-THYpe have been validated, each in a single study, but not independently. ThyGenX/ThyraMIR, a combination of the 7-Gene Panel with a miR expression classifier, achieved a sensitivity of 94%, specificity of 80%, NPV of 97%, and PPV of 68% (34). The RosettaGX Reveal has shown an NPV of 92%, PPV of 43%, sensitivity of 74%, and specificity of 74% (35). The ThyroPrint, a 10-gene classifier, has shown an NPV of 98%, PPV of 78%, sensitivity of 93%, and specificity of 81% (36). Mir-THYpe, a Brazilian 11 miRNA expression classifier, has shown an NPV of 96%, PPV of 76%, sensitivity of 95%, and specificity of 81% (37).

**Figure 3:**
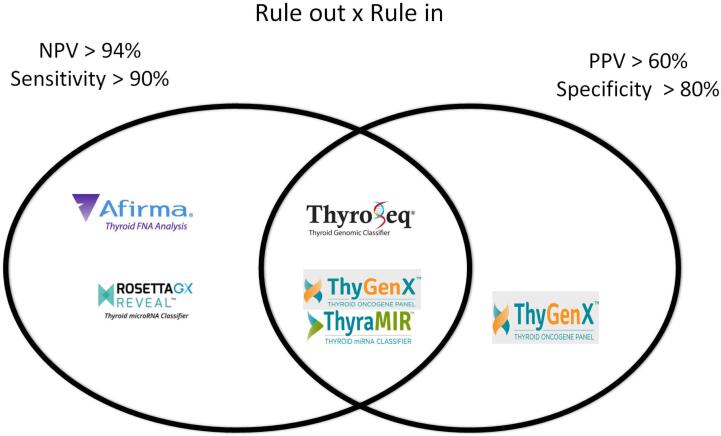
Commercially available rule-out and rule-in tests. Afirma-GEC and RosettaGX Reveal could be classified as rule-out tests (based on original publication data), ThyGenX alone, as a rule-in test, and ThyroSeq v3 and ThyGenX + ThyraMIR as both, rule-in and rule-out tests.

Based on these data, the available tests can be classified as either a “rule-in” or “rule-out” test (Figure 3). Specifically, when the diagnostic test is intended to predict benign nodules (rule out), it will require a high NPV, while when intended to predict malignancy (rule in), it will require a high PPV. According to Vargas-Salas and cols. (38), in order to consider a test as having good rule-out ability, the test should have an NPV of at least 94%; this means that a residual risk of malignancy would be lower than 6% for a negative result, which should be close to a Bethesda II cytology. A minimum sensitivity of > 90% is necessary to keep the NPV above 94% in a broad range of disease prevalences. There is no consensus on the minimum required PPV to consider a rule-in test to be good; however, a specificity rate above 80% would result in a PPV above 60% for a disease with a prevalence rate above 25%.

### Are the current tests helpful enough in diagnosing indeterminate thyroid FNAB samples?

If we consider the NPV, PPV, sensitivity, and specificity rates mentioned before, the currently available molecular tests would be considered as either rule-in or rule-out tests, as shown in Figure 3. In detail, the Afirma-GEC and RosettaGX Reveal could be classified as rule-out tests (based on original publication data), ThyGenX alone as a rule-in test, and ThyroSeq v2 and ThyGenX + ThyraMIR as both, rule-in and rule-out tests.

Knowing the pretest malignancy risk at each test location is important to estimate the actual NPV, as discussed above; this is true particularly for rule-out tests. The pretest malignancy risk is institution-dependent and requires knowledge of the risk of malignancy for each Bethesda category (38,39). However, in daily routine, patients often bring FNAB results from various laboratories; thus, it is almost impossible to know the pretest malignancy risk for every single lab, making it hard to judge a benign test outcome of a rule-out test since the real NPV is unknown. This problem can lead to an incorrect test interpretation and wrong surgical referral. Therefore, before a molecular test is recommended to a patient, the pretest malignancy risk should be determined, and the assessment should also include other features to refine the risk level, such as ultrasound features (hypoechoic solid nodules, microcalcifications, and irregular borders) (40), cytological features (nuclear atypia, which increases the risk of malignancy) (41), and the patient's history of radiation exposure, preferences, and family history of thyroid cancer.

Further limitations of the commercially available tests must be considered. For the rule-out tests: (a) none of the validation studies (33) could confirm the initial sensitivity and specificity of the test; (b) benign samples are often not resected, thus, the number of false-negative test outcomes are most likely underestimated and, for clinical practice, are currently unknown (42); and (c) HC tumors remain one of the main causes of incorrect tests. On the other hand, rule-in tests, in my opinion, can be somewhat a double-edged sword, since the correlation between the presence of mutations and malignancy is still imprecise in some cases.

Some mutations, like BRAF V600E and TERT, are highly specific and have been well studied, showing almost a 100% risk of PTC (43,44). However, the impact of preoperatively detecting *RAS* mutations or *PAX8/PPAR*γ fusions is still evolving. According to Nishino and cols., the PPVs of *RAS* and *PAX8/PPAR*γ for positive test outcomes among cytologically indeterminate aspirates can vary from 57% to 100% (42). Similarly, in a systematic review by Sahli and cols. including 8,162 patients, *RAS* mutations and *RET/PTC* and *PAX8/PPAR*γ rearrangements were detected in up to 48%, 68%, and 55% of all benign nodules, respectively. Moreover, some malignant lesions showed no mutations at all (33).

Since *RAS* mutations can be present in 30–45% of the FTCs, 30–45% of the follicular variant PTCs, 20–40% of the poorly DTCs, 10–20% of the anaplastic thyroid cancers, and 20– 25% of the FAs, their identification in molecular tests can be more confusing than helpful (45).

Xing, in an excellent review, showed us the light at the end of the tunnel (45). According to his findings, RAS mutations used alone have low diagnostic sensitivity and specificity, since histologically benign nodules can be conservatively managed for a long term. However, even when histologically confirmed to be malignant, tumors positive for a *RAS* mutation have limited aggressiveness. Thus, based on the findings of Medici and cols. (46), Xing suggests that coexistence of a *RAS* mutation with additional oncogenic changes should be treated differently than the finding of a *RAS* mutation alone in terms of prognostic significance. If only *RAS* mutations are found in a DTC, it is reasonable and safe to assume a good prognosis.

Therefore, there are still important technical limitations, as well as limitations with regard to the knowledge about the relevance of single mutations, since it seems that thyroid cancer is unlikely to be accounted for by the effects of a small number of genes but by a complex interaction of multiple factors (47,48). However, with improvements in the tests, these limitations should be minimized.

The technology in the new Afirma test has changed: GSC is now used, instead of GEC, in order to gain specificity and avoid histologically benign samples to be classified as suspicious. The test uses RNA-based NGS to measure gene expression, sequencing of nuclear and mitochondrial RNAs, changes in genomic copy number including loss of heterozygosity, and the development of enhanced bioinformatics and machine learning strategies. The initial validation study of the Afirma test has shown a 36% increase in specificity compared with the GEC (47). Translating into practice, at least one-third of the Bethesda III and IV nodules that are histopathologically benign will receive a benign result using the GSC compared with the GEC. The expectation is that with the 68.3% gain in specificity with the GSC, testing of Bethesda III and IV and HC neoplasms may safely reduce unnecessary surgeries.

New information has also been added to ThyroSeq v3 in order to improve sensitivity. The existing test has been expanded with the incorporation of recently discovered molecular markers (new driver mutations and gene fusions), copy number variations and, also, improvement of sequencing assays, allowing the detection of multiple and various types of genetic alterations with a limited amount of cells (49). Moreover, the test's accuracy for detecting various types of HC tumors has improved. The first results are very promising: Nikiforova and cols. demonstrated a sensitivity of 94% in the training set and 98% in the validation set (Figure 2); however, an independent, prospective, multicenter study is still needed.

Despite the known limitations described here, these tests seem to be moving toward an important improvement. Still, independent validation studies of the new tests are urgently needed. Moreover, we are still waiting for solutions for other problems: high costs and challenges to send samples for analysis, especially in countries outside the Europe-US axis. In Brazil, the cost of a molecular test is almost as high as the cost of a total thyroidectomy. In addition to pricey molecular tests, import and export taxes, shipment costs, and daily variations in the dollar exchange rate have to be considered. Moreover, an excessive bureaucracy restricts the offer of the tests to one laboratory in Brazil. Patients interested in molecular testing have to travel to this lab to collect FNAB material. Moreover, none of the health insurances cover the costs of the tests. Thus, the application of commercial molecular tests to improve the diagnosis of cytologically indeterminate thyroid FNABs is still far from our daily clinical practice.

### The Brazilian experience with molecular tests

Although diagnostic parameters of sensitivity, specificity, PPV, and NPV have been published for the different available tests, each institution is required to determine its own risks of malignancy for the different indeterminate categories, particularly in regard to PPV and NPV. Only based on these local malignancy rates can a test be defined as being good or not in terms of local PPVs and NPVs. Based on that, I conducted a survey of all tests performed in Brazil using commercially available assays to evaluate their usefulness and performance as rule-in and rule-out tests in the country. The results of the survey are detailed below.

Molecular tests have been available in Brazil since 2013. The first commercialized test was miRInform, and a total of seven tests were performed in the country before the test was replaced with ThyGenX. The ThyGenX/ThyraMIR was commercialized for a short period of time, and only three ThyGenX tests were performed.

Currently, only the ThyroSeq v2 (and now the ThyroSeq v3) test is commercialized in Brazil (by the laboratory Cytolog), since the RosettaGX Reveal has been recently removed from the market. Overall, 13 ThyroSeq v2, 4 ThyroSeq v3, and 5 RosettaGX Reveal tests have been performed (Table 1). The Afirma test was previously commercialized by the laboratory Fleury but is no longer available. Because thyroid nodules with mutation-negative (ThyroSeq v2 and v3) or benign results when tested with Afirma-GEC and RosettaGX Reveal are rarely resected, the false-negative rates and the sensitivity and specificity values of these tests in our statistics are currently unknown. Of all, 8 out of 17 samples analyzed with either the ThyroSeq v2 or the ThyroSeq v3 showed a positive result (presence of mutation); on histology, 4 of these samples with a positive result confirmed to be malignant on histology, while 2 were benign (1 was an FA with a *THADA/IGF2BP3* fusion, and 1 was negative for malignancy and had associated Hashimoto's thyroiditis with the presence of *MET* gene overexpression) and 2 have still not been operated on. Of the 5 samples evaluated with the RosettaGX Reveal, 3 were suspect for malignancy (1 was confirmed be malignant on histology), while 2 have not been operated yet.

**Table 1:** Description of all molecular tests commercially available in Brazil and performed to the current date to evaluate nodules deemed cytologically indeterminate on fine-needle aspiration biopsy

Test	Result	Bethesda	Histology
ThyroSeq v2	Positive (*THADA/IGF2BP3* fusion)	SFN (Bethesda IV)	Follicular adenoma
ThyroSeq v2	Positive (NRAS + TERT)	AUS (Bethesda III)	Papillary carcinoma
ThyroSeq v2	Positive (multiple gene expression with abnormalities)	SFN + HC (Bethesda IV)	Papillary carcinoma, oncocytic variant
ThyroSeq v2	Positive (BRAF K601)	AUS	Papillary carcinoma, follicular variant
ThyroSeq v2	Positive (overexpression of the *MET* gene)	AUS	Negative for malignancy, Hashimoto's thyroiditis
ThyroSeq v2	Positive (overexpression of the *MET* gene)	AUS	NO
ThyroSeq v2	7 negative	4 AUS / 3 SFN	NO
ThyroSeq v3	Positive (KRAS + copy number + gene expression alteration)	SFN + HC (Bethesda IV)	Multifocal micropapillary carcinoma
ThyroSeq v3	Positive (copy number alterations)	SFN + HC (Bethesda IV)	NO
ThyroSeq v3	2 negative	1 AUS / 1 SFN	NO
RosettaGX Reveal	Suspect	SFN	Papillary carcinoma, solid variant
RosettaGX Reveal	2 suspect	2 SFN	NO
RosettaGX Reveal	2 benign	1 AUS / 1 SFN + HC	NO

SFN: suspicious for follicular neoplasm; AUS: atypia of undetermined significance; HC: Hürthle cell tumor; NO: not operated.

Unfortunately, the Afirma-GEC data for the Brazilian population was not accessible due to the ethical policy of the Fleury laboratory.

Due to limited follow-up data, the sensitivity, specificity, and accuracy of these tests could not be determined for the Brazilian population and still remain to be evaluated. Moreover, as suggested by different groups, it is strictly important for each local setting to assess their own pretest probability of malignancy. As mentioned earlier, although these molecular tests are available in Brazil, due to their high costs, they still have a limited applicability in daily clinical practice.

The Mir-THYpe test was developed and validated by the startup ONKOS Diagnósticos Moleculares LTDA in partnership with the Cancer Hospital of Barretos. This is the only genetic test for molecular classification of indeterminate thyroid nodules that has been developed and validated exclusively in Brazilian patients. The Mir-THYpe analyzes genetic material (miRs) extracted from previously collected FNAB slides. The results of the Mir-THYpe validation study showed an NPV of 96%, PPV of 76%, sensitivity of 94.6%, and specificity of 81% (37), within a range of pretest probability of malignancy of 39%. With these numbers, the test showed to have both optimal rule-in and rule-out performances and to be safe in all three indeterminate categories: III (AUS/FLUS), IV (suspicious for follicular neoplasm), and V (suspicious for malignancy). These are promising data, especially for our population. However, a multicenter, independent study and longitudinal follow-up studies of unresected nodules with negative molecular testing results are strongly recommended.

In conclusion, while some tests have been withdrawn from the market, it seems that Afirma-GSC and ThyroSeq v3 are well-established tests that can be used as another tool in addition to clinical, radiological, and cytological features to help diagnose indeterminate FNAB samples. The initial sensitivity and specificity rates of the previous versions of the tests lack confirmation, and the tests underestimate false-negative cases; additionally, HC tumors remain one of the major causes of incorrect tests. Moreover, some somatic mutations still need more clarification in regard to their functional characterization and impact on tumor aggressiveness. The new versions of both tests, Afirma-GSC and ThyroSeq v3, show improved sensitivity and specificity but still require further independent validation. Still, local malignancy rates need to be determined, and more independent validation studies must be performed, in addition to studies comparing the performance of each test, although this may be difficult due to the individual characteristics and objectives of each test. Finally, we are still waiting for validation results of new tests (ThyroPrint and Mir-THYpe), since these tests have a much lower cost and, therefore, will be more accessible to patients in Latin America and Brazil.
